# Ecological niche measurement and high-quality development of "the Belt and Road" core area

**DOI:** 10.1371/journal.pone.0302550

**Published:** 2024-05-13

**Authors:** Hang Zhang, Nurguli Abdusuli

**Affiliations:** School of Economics and Management, Xinjiang Agricultural University, Urumqi, China; Feroze Gandhi Degree College, INDIA

## Abstract

A new stage in promoting the construction of the Silk Road Economic Belt Core Area, and Xinjiang has been transformed from a relatively closed inland area into an open border. In order to promote the high-quality development of Southern Xinjiang and solve the imbalance contradiction between the development of the Northern Xinjiang and Southern Xinjiang, taking the four districts in Southern Xinjiang as the study area, constructing a high-quality development ecological niche index system of three levels, namely economic, social and ecological, adopting the entropy method to assign weights to the evaluation indexes, and measuring the ecological niche width and the degree of ecological niche overlap of this region in the period from 2011 to 2020. The results show that: Firstly, tourism has the greatest impact on the ecological niche of economic development in state N, with a weighting of 14.18%; Secondly, the ecological status width of economic development in state N demonstrates a structural characteristic of “low level and low gap”. The average value of ecological niche width is at class III, indicating a low development status and weak regional influence; Thirdly, the ecological niche overlap of state N is significantly influenced by spatial factors. Regions Z and S are closer together, resulting in higher competition for resource utilization and an average ecological niche overlap at class II. The other two regions are at class III. According to the theory of ecological niche expansion and separation, a specialization separation strategy should be adopted for areas with "low width and high overlap", and a strengthening expansion strategy should be adopted for areas with "low width and low overlap", to optimize the structure of ecological niches and promote high-quality development of the region.

## 1. Introduction

Since the proposal of the Silk Road Economic Belt 10 years ago, Xinjiang has demonstrated its irreplaceable status and role in its construction through practical contributions. On 26 August 2023, Xi Jinping emphasized the importance of fully utilizing Xinjiang’s unique location advantages, actively integrating into the new development pattern, and accelerating the construction of the core area of “the Belt and Road” from a practical perspective after listening to a report on Xinjiang’s work. With the promotion of the Belt and Road initiative, Xinjiang has transitioned from a relatively closed inland region to an open frontier [1]. However, the high-quality development of Xinjiang has been hindered by the problem of development imbalance and incoherence between the north and south borders [2]. While acknowledging the disparity between the overall economic development of Southern Xinjiang and its regional volume, it is important to note that the region is abundant in energy resources, boasts unique location advantages, and has significant potential for special industries [3]. Additionally, it has clear advantages in providing aid to Xinjiang and possesses the necessary foundation, conditions, potential, and advantages to achieve high-quality development. Therefore, based on the current development trends, the Southern Xinjiang region needs to clarify its development status, assess resource utilization, and provide theoretical support to narrow the gap between Northern and Southern Xinjiang and achieve high-quality development.

Regional economic development has been assessed as a multi-level comprehensive assessment in existing studies. Early economic research in China was mostly used to study the speed of economic development. With the guidance of the green development concept, ecological civilization construction and other policies, China is now in a transition period from high-speed development to high-quality development [4–7]. High-speed economic growth does not fully represent high-quality economic development, and short-term high-speed growth may cause social problems, such as resource or environmental problems [8], and the intensification of polarisation between the rich and the poor [9]. It therefore focuses on the greening of the economy [10–15] or sustainable development [16–20] now. These are included under the heading of high-quality development. The prevailing view is that the development is a complex system. Regional economic development is influenced by a variety of factors and is a comprehensive expression of the economy, society and ecology of a region [21–23]. No single indicator can fully reflect the level of economic development, it should be scaled with multi-level indicators that meet the needs of the assessment. Accordingly, most of the current studies are comprehensive assessments of the level of regional economic development, which are not limited to economic indicators, but also include indicators measuring demographic, social, ecological and environmental aspects [15, 24–30]. Based on the continuous deepening of research on regional economic development, scholars believe that economic development is a continuous evolutionary process from low to high level and from quantitative change to qualitative change [31]. During the evaluation process, it is essential to consider both "state" and "trend" dimensions for accurate analysis. It is aligning with the theory of ecological niche, which comprehensively examines the evaluated object through the integration of its states and trends [32]. The niche ecostate-ecorole theory fit these dual dimensions well, so some scholars apply ecological niche theory in the study of regional economy to the evaluation and analysis of the level of regional economic development, the competitiveness of regional economy and regional competitions and partnerships, etc. These studies have mainly focused on coastal or inland areas, while there are very few ecological niche assessments of economic development for border areas.

**Fig 1:**
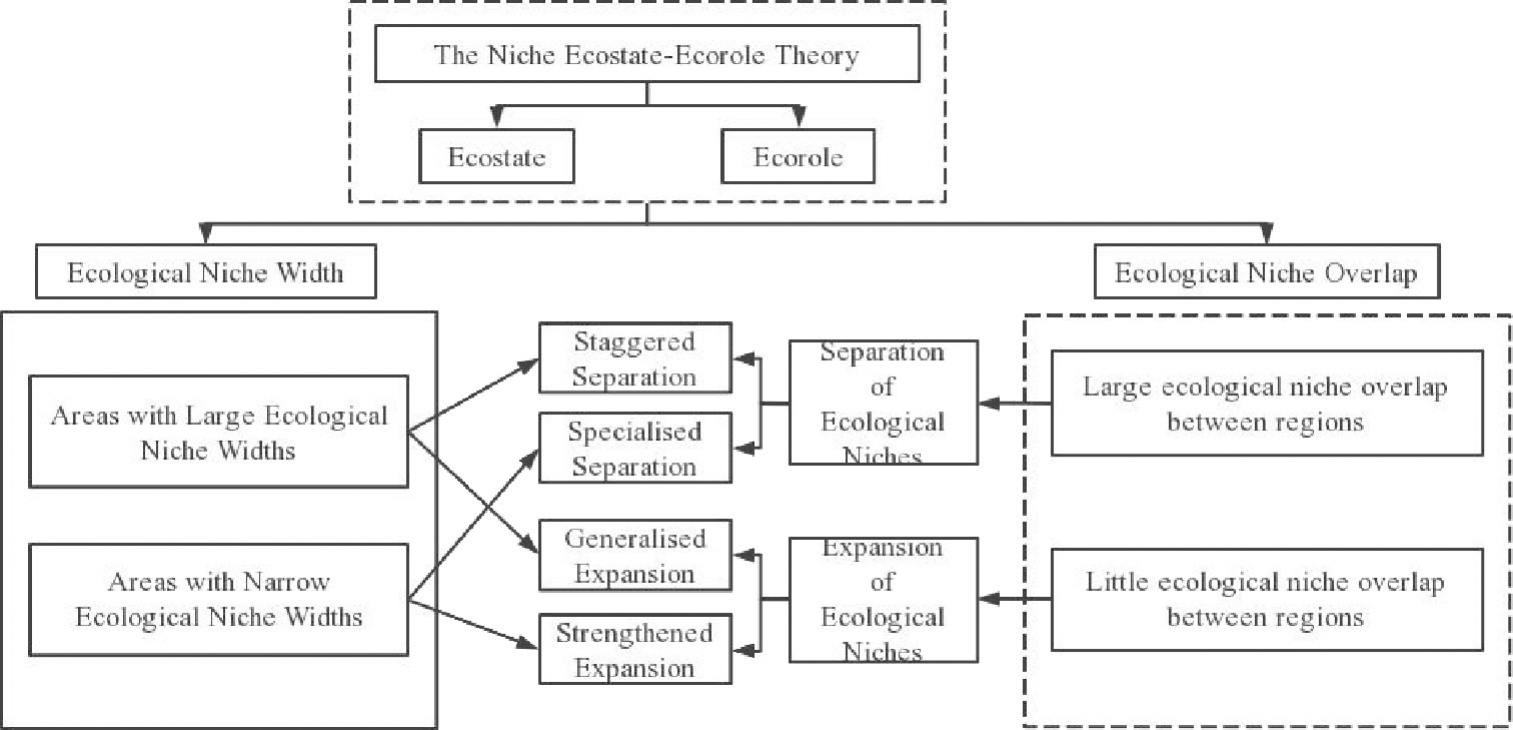
Ecological niche theory relationship diagram.

The concept of the ecological niche comes from ecology, first defined by Joseph Grinell [33] in his study of organisms in nature, and since then foreign scientists have continued to add to its meaning [34, 35]. After almost a century of evolution, the notion of the "ecological niche" now exceeds the scope of ecological studies and is being progressively extended to other domains including economic [36–38], tourism [39–41], cultural [42–44], technological [45, 46], and industrial [47] niches. The application of ecological niche theory in the field of regional economics is relatively recent, but the model of analogy between regional units and species units has been widely used in the study of regional geography and regional economics [48–53]. The ecological niche theory is applied to the study of economic development evaluation and the ecological niche concept of economic development is defined as: the spatial and temporal space occupied by different ecological units in the ecological system of regional economic development under the influence of many factors such as social, economic and environmental factors, as well as the relative status and role of that ecological unit in relation to other ecological units. The ecological niche theory encompasses primarily the ecological niche ecostate-ecorole, the ecological niche width, the ecological niche overlap, the expansion and separation of ecological niche, the relationship of these theories is shown in Fig 1.

Regional economic development is a cohesive entity that arises from the interplay between internal and external factors associated with economic growth in a specific locality, either directly or indirectly [54]. From a multi-dimensional spatial and temporal perspective, the regional economic development system is a complex ecosystem with multiple relationships, dimensions and levels. The development process itself moves from low to high levels and from quantitative change to qualitative change [6, 7]. Therefore, when assessing the progress of regional economic development, it is crucial to consider both the "state" and "process" aspects, in line with the niche ecostate-ecorole theory. Using the theory of ecological niche to assess economic development, the term "ecostate" describes the present state of regional economic development, while "ecorole" refers to the rate of renewal and growth, indicating a trend towards change.

Ecological niche width and degree of overlap are quantitative expressions of the position or degree of competition of each unit in the regional economy, based on the concepts of "ecostate" and "ecorole" of ecological niches. On that basis, the calculation of ecological niche width is applied to the study of economic development, and the larger the ecological niche width of an ecological unit, the higher the position of the ecological unit in the ecosystem of economic development [55]. Ecological niche overlap is the root cause of competition between ecological units [56], and refers to the phenomenon in an ecosystem where two ecological units with the same or similar ecological niche compete for the same resources in the same space [57]. A quantitative study of the degree of competition for economic development between regions, reflecting the competition for resources within the regional economic development system by calculating ecological niche overlap between different regions.

Ecological niche expansion or separation is a strategy for regions to adjust their ecological niche width and overlap characteristics, promoting sustainable economic development through various means. Ecological niche expansion in economic development is the process of increasing regional competitiveness and dominance to improve or maintain economic development within the current conditions of the regional economic system, achieved by expanding the region’s ecological niche. A minor overlap within a region is more probable to lead to ecological niche expansion. This is due to inefficient usage of economic development resources, requiring ecological niche expansion. This is manifested in two ways. First, generalized expansion. The higher ecological niche width for economic development means that the economic development of the region is more mature, and the resources available for development in the region are limited, so the ecological units in the system evolve towards "omnivory" or "generalization" in order to break the bottleneck of economic development [58]. Second, strengthened expansion. Since economic development is relatively weak in areas with small ecological niche width, and the small width of economic development limits the availability of various resources within the region, it is necessary to increase the level of regional economic development by exploring potential ecological niches or introducing new ecological niches. Ecological niche separation refers to differences in the selection of ecological niches across different regions to decrease competition for resources in numerous dimensions [59]. Ecological niche separation arises in regions where there is substantial overlap, and considerable overlap in economic niche indicates similarities in resource use or development across various parts of the area. This competition for resources and regions’ exclusion suggest a requisite for ecological niche separation. There are two specific scenarios. First, specialized separation. Areas with narrower ecological niches have a less favourable foundation for economic advancement and hold a lower position within the regional economic development scheme. To prevent the undue expansion of ecological niches in other regions placing significant pressure on its own economic development, it is imperative to concentrate on the growth of its own favourable projects to sidestep direct competition with other regions. Second, staggered separation. Regions with broad ecological niche widths possess a strong potential for economic growth and ample resources accessible through the regional economic development system. Although there is competition present, ecological niches can be dislocated by distinguishing spatial, temporal or resource-use niches, resulting in a complementary economic development approach rather than specialization.

Based on the characteristics outlined above, ecological niche theory can combine both the current state and trend to analyse the level of economic development. Additionally, its rigorous theoretical framework can propose appropriate ecological niche strategies for the characteristics of different regions. Currently, the core area of the Belt and Road is experiencing rapid development, and the ecological niche perspective is well-suited for assessing local economic development. Therefore, this paper takes the state N in South Xinjiang as a case study and applies the ecological niche theory to evaluate the economic development of this area. The state N is regarded as an economic ecosystem, and the regions K, Z, S and T are regarded as ecological units in the system, and the ecological niche widths and overlapping degrees of the four regions are calculated by the ecological niche model, so as to evaluate the status of each region and the degree of competition in the state N, so as to provide theoretical references and data support for the economic development of the state N.

## 2. Materials and methods

### 2.1. Ecological niche model

#### 2.1.1. The ecological niche width model

The formula for calculating the combined width of the ecological niche of economic development is:

$$M_{i}=\frac{\sum_{\alpha =1}^{m}N_{i\alpha }w_{\alpha }}{\sum_{\alpha =1}^{m}w_{\alpha }},$$ (1)

in Eq(1): α = 1,2,…,m denotes the number of indicators, M_i_ denotes the ecological niche width of economic development of the ecological unit, and w_α_ denotes the weight of each indicator variable, the magnitude of which reflects the degree of influence of each indicator factor on the ecological niche of economic development, and can reflect the degree of importance of each indicator factor. The ecological niche width for a single metric (N_iα_) is calculated using the formula:

$$N_{i\alpha }=\frac{\left(S_{i\alpha }+AR_{i\alpha }\right)}{\sum_{i=1}^{n}\left(S_{i\alpha }+AR_{i\alpha }\right)},$$ (2)

in Eq (2): i = 1,2,…,n denotes the number of ecological units, and N_iα_ denotes the width of the ecological niche of the ecological unit i on the indicator α; S_iα_ and R_iα_ represent the ecostate value and ecorole value of the ecological unit on the indicator, respectively, where the ecostate value is the data after the current data of each indicator is dimensionless, and the ecorole value is the data after the growth rate of each indicator is dimensionless; A is the scale conversion factor, which is taken as 1 because the time span for calculating the growth rate is 1 year. The interval of the ecological niche width is [0, 1], and the closer it is to 1, the higher the economic development status of the ecological unit and the greater its regional economic influence or dominance, and vice versa.

#### 2.1.2. The ecological niche overlap model

The formula for calculating the degree of overlap of ecological niches between two regions is as follows:

$$b_{ij}=\frac{\sum_{\alpha =1}^{m}N_{i\alpha }N_{j\alpha }}{\sqrt{\sum_{\alpha =1}^{m}N_{i\alpha }^{2}\sum_{\beta =1}^{m}N_{j\alpha }^{2}}},$$ (3)

in Eq (3): the ecological niche overlap between areas i and j can be denoted by b_ij_; the ecological niche widths of areas i and j on indicator α can be represented by N_iα_ and N_jα_, respectively, whilst d indicates the straight-line distance between the two areas’ spatial centres. The range for the ecological niche overlap is [0, 1], with 0 indicating complete ecological niche separation and 1 indicating complete ecological niche congruence.

In addition, based on the principle of distance decay, this paper posits that the level of overlap between regions is influenced by the distance separating the two entities involved. The greater the distance between two regions, the less likely they are to compete for the same resource. Therefore, spatial factors need to be taken into account when calculating the ecological niches overlap for economic development. The gravitational model is introduced in order to adjust the extent of overlap among ecological niches such that it aligns more accurately with practical economic principles.

$$B_{ij}=G\frac{b_{ij}}{d_{ij}},$$ (4)

in Eq (4): B_ij_ represents the improved ecological niche overlap, while d_ij_ indicates the linear distance between the area i and j. G is a fixed parameter set to 1.

This paper assumes that third parties do not affect the degree of overlap between the two regions. Therefore, a mean method is utilized to compute the collective overlap of the ecological niches for economic progress in the area:

$$\delta_{i}=\frac{\sum_{j=2}^{n}B_{ij}}{n-1},$$ (5)

in Eq (5): δ_i_ represents the degree of overlap of the region’s integrated ecological niche for economic development.

### 2.2. Establish an evaluation index system for ecological niche of economic development

#### 2.2.1. Selection of indicators

Regional economic development aims to achieve coordinated growth across the economic, social, and ecological dimensions, in order to fulfil the public’s desire for an improved quality of life. In the economic dimension, high-quality development should be dynamic and sustainable, not only in terms of steady growth in economic output, but also in terms of sustainable optimization of the economic structure and the emergence of new industries. In the social dimension, high-quality development ought to facilitate the populace’s enjoyment of development benefits, foster social justice on the premise of people’s living standards’ continual betterment, and reduce the urban-rural disparities. In the ecological dimension, the concept of green development and the synergistic development of economy and ecology should be fully reflected in order to achieve high-quality development, which includes reducing pollution, lowering energy consumption and creating an ecological environment worth living in. Therefore, this paper presents a summary of the indicator system used to evaluate China’s recent economic development. It selects commonly-represented indicators and measures the overall strength of regional economic development in state N from three perspectives: economic, social, and ecological. Subsequently, it identifies 26 indicators, taking into account data accessibility, and details them in Table 1.

**Table 1:** Ecological niche evaluation system for economic development.

System Layer	Normative Layer	Indicator Layer	Nature of Indicators	Description of Indicators
Economic Subsystem	economic aggregate	total regional production	+	Measuring the total level of the economy.
		fixed-asset investment	+	Measuring the level of social investment.
		unit yield of food crops	+	Measuring the level of agricultural development.
		industrial output per capita	+	Measuring the level of industrial development.
		tourism revenue	+	Measuring the level of tourism development.
	economic structure	share of tertiary output in total production	+	Measuring industrial structure.
		total retail sales of consumer goods as a share of total production	+	Measuring the distribution structure.
		percentage of population in agriculture	-	Measuring labour force structure.
Social Subsystem	living standards of the population	disposable income of the population	+	Measuring the level of income of the population.
		unemployment rate	-	Measuring the employment status of the population.
		pension insurance participation rate	+	Measurement of the population’s insurance status.
		health insurance participation rate	+	
	population	total population at year end	+	Measuring total population.
		population density	-	Measuring population congestion.
	healthcare	investment in health	+	Measuring the scale of health development.
		number of beds in health-care institutions per 10,000 persons	+	Measuring healthcare resources.
		health technicians per 10,000 population	+	
	education	investment in education	+	Measuring the scale of educational development.
		number of students enrolled in general higher education	+	Measuring the level of education.
		teacher-student ratio	+	Measuring educational resources.
Ecological Subsystem	environmental carrying capacity	cropland area	+	Measuring arable land resources.
		proportion of days with satisfactory air quality	+	Measuring air quality.
		fertilizer use per unit of arable land area	-	Measuring the quality of arable land.
	energy consumption	water use per 10,000 yuan of total production	-	Measuring water consumption.
		energy consumption per 10,000 yuan of industrial output	-	Measuring energy consumption.
		electricity consumption per 10,000 yuan of industrial output	-	Measuring electricity consumption.

Note

“+” represents a positive indicator, “-” represents a negative indicator.

#### 2.2.2. Allocation of indicator weights

The entropy value method provides an effective means of dealing with multiple indicators, uncertainty information, and broad applicability, making it an accessible and practical approach. Thus, this paper applies the entropy value method to weight indicators of ecological niche of economic development. The raw data for the indicators were first standardized by the extreme value method:

$$r=\frac{x-x_{\min}}{x_{\max}-x_{\min}},$$ (6)

in Eq (6): r represents the standardized processed value, whilst x represents the original data, x_min_ denotes the minimum value within the dataset, and x_max_ denotes the maximum value within the dataset. Next, calculate the entropy of the indicator:

$$e_{\alpha }=\frac{1}{\ln\;n}\sum_{i=1}^{n}p_{i}\;\ln\;p_{i},$$ (7)

in Eq (7): e_α_ denotes the proportion of data processed using entropy within this data set, whilst p_i_ represents the entropy of the initial indicator. Finalize the weights

$$w_{\alpha }=\frac{1-e_{\alpha }}{\sum_{\alpha =1}^{m}\left(1-e_{\alpha }\right)},$$ (8)

in Eq (8): the final weight of each indicator variable is represented by w_α_.

#### 2.2.3. Data sources and processing

The data presented in this paper are sourced from the Statistical Yearbook of Province X for 2011–2021, the Sixth and Seventh National Population Census Bulletin of Province X, and the 2010–2020 Statistical Bulletin of National Economic Development for State N. Missing data exhibiting significant monotonicity were supplemented using linear interpolation, while irregular data were supplemented by averaging. Finally, the Stata software was utilized to calculate weightings for the indicators, and Table 2 displays the results. In addition, the calculation of the ecological niche overlap involved using ArcGIS software to identify the spatial centre coordinates of the four regions, straight-line distances were then determined between these regions. As area Z is the nearest to area S, this distance was used as the unit of measurement to determine the relative distances between the other areas.

**Table 2:** Allocation of weights in the ecological niche evaluation system for economic development.

System Layer	Normative Layer	Indicator Layer	Indicator Weights
Economic Subsystem	economic aggregate	total regional production	0.0166
		fixed-asset investment	0.0103
		unit yield of food crops	0.0488
		industrial output per capita	0.0678
		tourism revenue	0.1418
	economic structure	share of tertiary output in total production	0.0098
		total retail sales of consumer goods as a share of total production	0.0390
		percentage of population in agriculture	0.0317
Social Subsystem	living standards of the population	disposable income of the population	0.0333
		unemployment rate	0.0115
		pension insurance participation rate	0.0200
		health insurance participation rate	0.0664
	population	total population at year end	0.0367
		population density	0.0221
	healthcare	investment in health	0.0511
		number of beds in health-care institutions per 10,000 persons	0.0698
		health technicians per 10,000 population	0.0258
	education	investment in education	0.0502
		number of students enrolled in general higher education	0.0565
		teacher-student ratio	0.0305
Ecological Subsystem	environmental carrying capacity	cropland area	0.0639
		proportion of days with satisfactory air quality	0.0254
		fertilizer use per unit of arable land area	0.0182
	energy consumption	water use per 10,000 yuan of total production	0.0344
		energy consumption per 10,000 yuan of industrial output	0.0138
		electricity consumption per 10,000 yuan of industrial output	0.0047

## 3. Results and discussion

### 3.1. Evaluation of ecological niche width for economic development

According to the constructed ecological niche evaluation system, the ecological niche width of each subsystem and the comprehensive ecological niche width in the four regions from 2011 to 2020 were calculated by Eqs (1) and (2), and the results are shown in Table 3.The findings indicate a fluctuating upward trend in the ecological niche widths across all four regions, with the most significant growth in region T, which increased by 120.3% between 2011 and 2020. Table 4 illustrates the division of regional grades into four levels based on the values of ecological niche width. The ecological niche width composite score for state N, from 2011 to 2020, was graded using the criteria specified. Regions K and S have comparable ecological niche widths and both will be designated as class II areas in 2020, an improvement from their previous class III status. Conversely, regions Z and T will be designated as class III areas in 2020, having previously been class IV areas between 2013 and 2016. Region Z was a class III area while region T was class IV in 2011 and 2012, which distinguishes them. The ecological niche widths of all regions, however, demonstrate significant improvement in 2020.

**Table 3:** Ecological niche widths of economic development subsystems in the four regions of the state N from 2011 to 2020.

Region	Year	Economic Subsystem	Social Subsystem	Ecological Subsystem	Synthesis
Region K	2020	0.7907	0.4242	0.6570	0.5956
	2019	0.3173	0.3724	0.5256	0.3768
	2018	0.2912	0.4097	0.6164	0.3995
	2017	0.2901	0.4088	0.6409	0.4026
	2016	0.2272	0.2002	0.3627	0.2361
	2015	0.2536	0.2080	0.4849	0.2691
	2014	0.2115	0.2275	0.4584	0.2587
	2013	0.2397	0.2182	0.3810	0.2522
	2012	0.2413	0.2023	0.5109	0.2661
	2011	0.4634	0.2105	0.5953	0.3647
Region Z	2020	0.2809	0.5704	0.3902	0.4356
	2019	0.2520	0.4035	0.4903	0.3620
	2018	0.1607	0.2787	0.4432	0.2619
	2017	0.2117	0.2947	0.5223	0.3009
	2016	0.1622	0.2409	0.3332	0.2269
	2015	0.1719	0.1973	0.4245	0.2244
	2014	0.1693	0.2421	0.2346	0.2142
	2013	0.2006	0.1895	0.2551	0.2041
	2012	0.2389	0.2209	0.4387	0.2624
	2011	0.2411	0.2339	0.3323	0.2523
Region S	2020	0.7188	0.5315	0.5506	0.6031
	2019	0.2181	0.4149	0.6012	0.3728
	2018	0.3990	0.3817	0.4837	0.4044
	2017	0.3378	0.4638	0.7944	0.4707
	2016	0.2335	0.2803	0.2914	0.2650
	2015	0.2447	0.2956	0.4134	0.2958
	2014	0.2078	0.2215	0.4774	0.2575
	2013	0.2530	0.2706	0.3109	0.2706
	2012	0.3024	0.2474	0.5112	0.3098
	2011	0.3573	0.2659	0.3499	0.3128
Region T	2020	0.4399	0.5553	0.4415	0.4948
	2019	0.1309	0.3651	0.4982	0.3008
	2018	0.1449	0.3342	0.4300	0.2803
	2017	0.1515	0.4679	0.5654	0.3678
	2016	0.0741	0.2952	0.2540	0.2077
	2015	0.0979	0.2365	0.2946	0.1951
	2014	0.0936	0.2126	0.2619	0.1770
	2013	0.1240	0.2577	0.2001	0.1996
	2012	0.1533	0.2401	0.3931	0.2329
	2011	0.1885	0.2359	0.2738	0.2246

**Table 4:** Ecological niche width classifications.

Class	Ecological Niche Width	Instruction
Ⅰ	0.75<M_i_≤1	The greater the value, the higher the economic development status of the region and the stronger its regional influence.
Ⅱ	0.5<M_i_≤0.75	
Ⅲ	0.25<M_i_≤0.5	
Ⅳ	0≤M_i_≤0.25	

The different dimensions of development in state N are graded through the evaluation of subsystems. The first aspect evaluated is the ecological niche width of the economic subsystems. Both the economic subsystems of region K and S have been unstable, fluctuating between class III and IV during 2011 and 2019. However, in 2020, the economic subsystem of region K reached class I, while the region S remained at class II. Compared to the first two regions, the economic subsystems of regions Z and T are relatively more stable, yet also comparatively underdeveloped, both classified as class IV until 2018. Region Z reached class III in 2019, while region T lagged behind by a year, reaching class III in 2020. Additionally, the ecological niche width of the social subsystem is assessed. Compared with the other three regions, the social subsystem of region S exhibits greater instability, oscillating between classes III and IV from 2011 to 2019. The social subsystem of region K is marginally inferior in comparison to the other three prefectures. In 2020, the ecological niche width of the social subsystems of the other three regions attains class II, while the region K remains at class III. Thirdly, this study evaluates the ecological niche width of different ecological subsystems. The ecological subsystems of state N display no consistent historical trends. In 2020, region K and S reached class II, while both region Z and T were at class III.

In short, between identical subsystems in different areas, as well as between different subsystems within the same region, there are differences in evolution of time trends. Overall, the ecological niche width of the four regions in state N is at a low level, with the combined economic development status of regions K and S slightly higher than that of regions Z and T.

Based on the average value of ecological niche width over a 10-year period, as presented in Table 5, the results indicate that the ecological niches have a comprehensive width with a structural characteristic of “low level and low gap”. The order of economic development from highest to lowest is region S, region K, region Z and region T. These four regions are all classified as class III, with low economic development status and weak regional influence and dominance. Starting from various subsystem perspectives, the ecological niche widths of the regions were ranked. The economic subsystems were ranked in descending order as region K, region S, region Z, and region T. The social subsystems were ranked in descending order as region S, region T, region K, and region Z. The ecological subsystem is ordered from highest to lowest as region K, region S, region Z, and region T. In conclusion, the level of regional economic development’s ecological niche width is affected comprehensively by the spatial and temporal correlation among the economic subsystems, social subsystems, and ecological subsystems. Region K’s ecological niche width for economic and ecological subsystems is slightly higher than that of region S. However, the lower ecological niche width of social subsystems in region K results in the ecological niche composite width being surpassed by region S.

**Table 5:** Mean ecological niche widths of economic development subsystems in the four regions of the state N.

Region	Region K	Region Z	Region S	Region T
Economic Subsystem	0.3326	0.2089	0.3272	0.1599
Social Subsystem	0.2882	0.2872	0.3373	0.3201
Ecological Subsystem	0.5233	0.3864	0.4784	0.3613
Synthesis	0.3421	0.2745	0.3563	0.2681

### 3.2. Evaluation of ecological niche overlap for economic development

According to the ecological niche assessment system, Eqs (3)–(5) were used to calculate the ecological niche overlap for economic development between each of the regions in State N from 2011 to 2020. The resulting data is presented in Table 6. Based on the value of the ecological niche overlap degree, the regional grade is classified into four levels, illustrated in Table 7. The findings indicate that economic development overlap between region Z and region S was the highest, measuring above 0.75 from 2011 to 2019, achieving a class Ⅰ competition ranking. The level of competition decreased in 2020, yet remained at the class Ⅱ ranking. The primary cause of this phenomenon is the substantial overlap in resource usage between Regions Z and S due to their geographic closeness. The competing level between other areas is generally low, usually ranking at class III or IV. In addition, with the exception of the slight increase in ecological niche overlap between region S and T over the 10-year period, the ecological niche overlap of all other regions has shown a fluctuating downward trend. This shows that the existence of the phenomenon of competition has been noted in the process of economic development, and that different regions have developed in different directions in order to avoid competition.

**Table 6:** Ecological niche overlap for economic development between the two regions of state N from 2011 to 2020.

Year	Region K and Z	Region K and S	Region K and T	Region Z and S	Region Z and T	Region S and T
2020	0.2791	0.3108	0.4618	0.6857	0.2922	0.4809
2019	0.3335	0.3379	0.2209	0.9336	0.2017	0.3983
2018	0.3304	0.2955	0.3746	0.8015	0.2750	0.5009
2017	0.3345	0.3154	0.4100	0.8940	0.3096	0.4989
2016	0.3292	0.3332	0.4187	0.9284	0.3168	0.4865
2015	0.3321	0.3601	0.3877	0.9286	0.2957	0.4144
2014	0.3299	0.3516	0.4434	0.9338	0.3224	0.4907
2013	0.3228	0.3481	0.4438	0.9816	0.3385	0.4954
2012	0.3157	0.3163	0.4353	0.9706	0.3437	0.4974
2011	0.3298	0.3760	0.4668	0.9717	0.3282	0.4760

**Table 7:** Ecological niche overlap classifications.

Class	Ecological Niche Overlap	Instruction
Ⅰ	0.75<M_i_≤1	The greater the value, the stronger the competition between regions.
Ⅱ	0.5<M_i_≤0.75	
Ⅲ	0.25<M_i_≤0.5	
Ⅳ	0≤M_i_≤0.25	

Based on Table 8, the different dimensions of development in state N are graded through the evaluation of subsystems. The first aspect evaluated is the ecological niche overlap of the economic subsystems. Between 2011 and 2020, the economic subsystem in region S fluctuated between class II and III. In contrast, the economic subsystems in the other three regions remained stable, all remaining at the same level over the ten-year period, with the difference that regions K and T remained at class III, while the region Z remained at class II. Additionally, the ecological niche overlap of the social subsystem is assessed. Between 2011 and 2020, the social subsystems in the region S were more unstable and had a higher degree of overlap compared to the other three regions, oscillating between class II and III. In contrast, the overlap of social subsystems in the other three regions remained at class III. Thirdly, this study evaluates the ecological niche overlap of different ecological subsystems. The ecological niche subsystem hierarchy is comparable to that of social subsystems. Region S is more unstable and has a higher degree of overlap, oscillating between class II and III from 2011 to 2020. The overlap of social subsystems in the other three regions is primarily maintained at class III, with only region Z at class II in 2011.

**Table 8:** Ecological niche overlaps of economic development subsystems in the four regions of the state N from 2011 to 2020.

Region	Year	Economic Subsystem	Social Subsystem	Ecological Subsystem	Synthesis
Region K	2020	0.3717	0.3599	0.3143	0.3506
	2019	0.2966	0.3547	0.3533	0.2975
	2018	0.3351	0.3494	0.3379	0.3335
	2017	0.3554	0.3619	0.3539	0.3533
	2016	0.3735	0.3423	0.3447	0.3603
	2015	0.3919	0.3468	0.2769	0.3600
	2014	0.3832	0.3589	0.3131	0.3750
	2013	0.3756	0.3630	0.3432	0.3716
	2012	0.3576	0.3634	0.3314	0.3558
	2011	0.3984	0.3705	0.3908	0.3908
Region Z	2020	0.4787	0.4332	0.4353	0.4190
	2019	0.4963	0.4587	0.4221	0.4896
	2018	0.4778	0.4381	0.4005	0.4689
	2017	0.5213	0.4279	0.3311	0.5127
	2016	0.5403	0.4101	0.4588	0.5248
	2015	0.5414	0.4132	0.3913	0.5188
	2014	0.5447	0.3919	0.4457	0.5287
	2013	0.5559	0.4331	0.4649	0.5476
	2012	0.5478	0.4608	0.4670	0.5433
	2011	0.5560	0.4531	0.5432	0.5432
Region S	2020	0.5251	0.5356	0.5056	0.4925
	2019	0.5625	0.5502	0.5055	0.5566
	2018	0.5386	0.5274	0.4686	0.5326
	2017	0.5755	0.5308	0.4562	0.5694
	2016	0.5972	0.4965	0.5549	0.5827
	2015	0.6012	0.5117	0.4294	0.5677
	2014	0.6073	0.4870	0.4890	0.5920
	2013	0.6172	0.5139	0.5020	0.6084
	2012	0.5996	0.5167	0.5202	0.5948
	2011	0.6189	0.5168	0.6079	0.6079
Region T	2020	0.4273	0.3918	0.3597	0.4116
	2019	0.2622	0.4114	0.4079	0.2737
	2018	0.3842	0.4022	0.3343	0.3835
	2017	0.4130	0.3944	0.3770	0.4061
	2016	0.4267	0.3641	0.3801	0.4074
	2015	0.4330	0.3926	0.2657	0.3659
	2014	0.4282	0.3979	0.3708	0.4188
	2013	0.4338	0.4052	0.3977	0.4259
	2012	0.4299	0.4069	0.3807	0.4255
	2011	0.4302	0.3938	0.4236	0.4236

Table 8 shows the combined overlap of the different regions. Notably, regions K and T display low overlap while the ecological niche’s comprehensive overlap during the 10-year period is at class III. Region Z displays high overlap from 2011 to 2017, with the ecological niche’s comprehensive overlap at class II, which declines to class III from 2018 to 2020. In contrast, region S demonstrates high overlap from 2011 to 2019, with the ecological niche’s comprehensive overlap at class II, which only reduces to class III in 2020.

Based on the average value of ecological niche overlap over a 10-year period, as presented in Table 9, the results indicate that the ecological niche overlap is significantly influenced by spatial factors, region Z and S have a high ecological niche overlap due to their close spatial proximity. The order of the competition degree in economic development, from low to high, is region K, region T, region Z, and region S. The ecological niche overlap is low in region K and T, at level III, as well as high in region Z and S, at level II, but the latter two are gradually moving towards lower overlap. Starting from various subsystem perspectives, the ecological niche widths of the regions were ranked. The rankings of ecological niche overlap for the subsystems were consistent with the combined ecological niche overlap ranking, which is region K, region T, region Z, and region S. However, the geographic proximity of regions Z and S results in excessive competition between them, which influences the combined ecological niche overlap of these regions.

**Table 9:** Mean ecological niche overlaps of economic development subsystems in the four regions of the state N.

Region	Region K	Region Z	Region S	Region T
Economic Subsystem	0.3639	0.5260	0.5843	0.4069
Social Subsystem	0.3571	0.4320	0.5187	0.3960
Ecological Subsystem	0.3360	0.4360	0.5039	0.3698
Synthesis	0.3548	0.5097	0.5705	0.3942

## 4. Conclusions and recommendations

### 4.1. Conclusions

Based on previous research into evaluating economic development, this paper presents a case study of the borderland state N. The study applies the ecological niche theory to measure the width and overlap of the economic development ecological niche of the four regions within it. The study then evaluates the economic development status of state N, as well as the level of competition. The main conclusions are as follows:

Firstly, tourism is the primary factor influencing the ecological niche of economic development of state N. Table 2 shows the weight allocation of each indicator in the economic development ecological niche index system, as determined by the entropy value method used in this paper. Only one indicator, tourism income, has a weight of more than 10%, reaching 14.18%, while the weights of the other indicators are all below 10%. Secondly, the economic development’s ecological niche width in state N exhibits the structural characteristics of “low level and low gap”. According to the ecological niche width model, the average ecological niche width value of economic development in each region is at class III, indicating a lower development status and weaker regional influence and dominance. The level of regional economic development’s ecological niche width is affected comprehensively by the spatial and temporal correlation among the economic subsystems, social subsystems, and ecological subsystems. Thirdly, the spatial factors greatly influence the degree of overlap of the economic development ecological niche of state N. The ecological niche overlap model was used to measure the degree of overlap between economic development in each region. The geographic proximity of regions Z and S results in excessive competition between them, which influences the combined ecological niche overlap of these regions.

**Fig 2:**
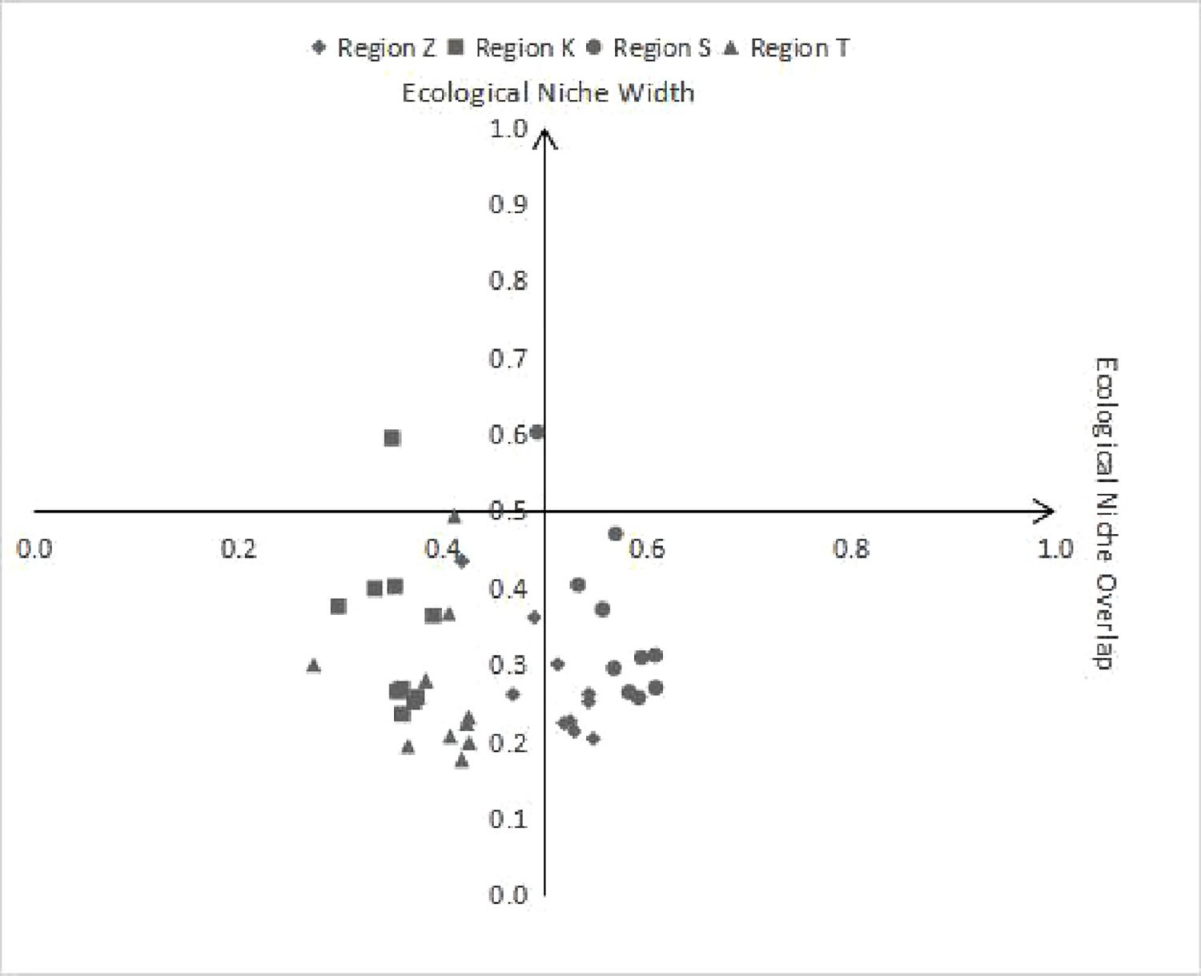
Scatterplot of width and overlap of ecological niches for economic development in the four regions of the state N.

To accurately portray the complete status of ecological niche width and overlap in each region of State N, a coordinate system is created with 0.5 as the dividing line. The ecological niche overlap serves as the horizontal axis, while the ecological niche width serves as the vertical axis. The figures of the ecological niche width and overlap of the four regions from 2011 to 2020 are marked on the graph to produce Fig 2. In terms of the combined ecological niche width and overlap, regions K and T exhibit a comparable economic development status, with most data points situated in quadrant 3, highlighting "low width and low overlap". Similarly, regions S and Z share a comparable economic development status, with most data points located in quadrant 4, denoting "low width and high overlap".

### 4.2. State N economic development strategy

The economic development of State N is classified, based on the ecological niche theory relationship shown in Fig 1. Different development strategies must be employed for distinct regions. Regions S and Z belong to the "low-width, high-overlap" region and call for a specialized separation strategy. On the other hand, regions K and T belong to the "low-width, low-overlap" field and require strengthened expansion tactics.

#### 4.2.1. Specialized separation

Due to the "low-width and high-overlap" characteristics, regions S and Z should implement ecological niche specialized separation in economic development. This requires these regions to leverage their local economic development advantages, strive to explore the region’s development potential, and achieve ecological niche separation from other regions through precise positioning. This will enable the implementation of a gap-type development strategy and create more space for the growth and survival of all stakeholders.

Region S is in the early stages of industrialization, with a weak industrial base. However, the region has the necessary conditions and resources for the development of new industries, such as oil and gas, green mining, new energy, and new materials. Therefore, Region S should leverage its location advantages and industrial base, expedite local oil and gas well exploration, support industrial park construction, promote comprehensive natural gas utilization, introduce and establish natural gas chemical industries, enhance new energy advantages, and accelerate the establishment of a modern industrial system. Furthermore, region S possesses abundant solar energy resources. Therefore, it is important to develop the photovoltaic industry and cultivate new forms of clean energy. This will promote the energy revolution and accelerate the planning and construction of a new energy system, ultimately reducing fossil fuel consumption and pollution. Region S has a high-quality new energy resource advantage, which serves as the foundation for the development of new energy-related industries. This strategy aims to achieve economic growth, reduce energy consumption, and decrease pollution. It is a specialized separation approach for the region S‘s development.

Region Z is geographically closer to region S and has a more mature agro-industrial system, but it is not as rich in new energy resources as region S. To reduce the overlap in resource use between the two regions, region Z can optimize its agro-industry, create a brand image for its speciality industries, and promote a high degree of agglomeration of factors of production in these industries to form an industrial and brand advantage. In addition, the integration of agriculture and tourism has extended the development of the agricultural industry beyond the primary sector to a more integrated approach involving both primary and tertiary industries. Therefore, region Z should integrate the agricultural speciality industry and tourism industry in an organic manner, and develop speciality agro-tourism products that are tailored to local conditions, in order to achieve a mutually beneficial outcome for both industries. Region Z has a development advantage in its mature agricultural industry system. The integration of agriculture and tourism is a key pathway for development, which can improve the efficiency of the agricultural industry and expand its development opportunities. This is achieved through the specialized separation strategy of region Z.

#### 4.2.2. Strengthened expansion

Regions K and T are characterized as having a "low-width, high-overlap" feature, and as such, they should implement the ecological niche strengthened expansion strategy in their economic development, with the main objective being the filling of shortfalls. This includes expanding potential ecological niches or introducing new ones.

In region K, the basic public service system needs improvement to strengthen the social subsystem. It is important to focus on promoting social security in the region. Improving healthcare and accelerating the construction of a high-quality education system are necessary steps to improve the ecological status of the social subsystem. A more proactive employment policy should also be implemented. In addition, promoting self-employment, attracting capital and talent from outside the region, and fostering entrepreneurship-led employment are important measures to enhance the ecological status of the social subsystem in region K by introducing new ecological niches. According to the above, the development of region K falls short in the area of social security. To improve the ecological status of region K, an strengthened expansion strategy is proposed, which includes expanding potential ecological niches and introducing new ones. The former involves improving medical care and education levels, while the latter aims to attract more population through policies.

Improving the economic development environment and optimizing the economic structure is an important step for region T to enhance its ecological position. Region T has a thriving agricultural economy, but its industrial sector lags behind, with a dominance of primary sector industries. Region T has a plentiful population, so the development of industries should focus on labour-intensive industries. Additionally, due to the region’s excellent agricultural resources, there is potential for the vigorous development of the deep processing industry of agricultural products. This can create a whole industrial chain pattern of agricultural product processing that integrates high-quality raw material bases, characteristics of industrial clusters, and productive service platforms. The result will be a green and organic agricultural product processing base. In addition, labour-intensive industries such as electronics assembly, textile and garment production, and ethnic handicrafts can also contribute to the development of the industrial economy in the region T. Based on the above, the economic shortcomings of the development of region T are primarily in the industrial sector. Given its abundant agricultural resources and labour force, prioritizing the development of labour-intensive industries could be an strengthened expansion strategy to enhance the region’s economy.

## Supporting information

S1 Dataset (XLSX)
